# AHRR cg05575921 methylation in relation to smoking and PM_2.5_ exposure among Taiwanese men and women

**DOI:** 10.1186/s13148-020-00908-3

**Published:** 2020-07-31

**Authors:** Disline Manli Tantoh, Ming-Chi Wu, Chun-Chao Chuang, Pei-Hsin Chen, Yeu Sheng Tyan, Oswald Ndi Nfor, Wen-Yu Lu, Yung-Po Liaw

**Affiliations:** 1 Department of Medical Imaging, Chung Shan Medical University Hospital, Taichung City, Taiwan; 2 Department of Public Health and Institute of Public Health, Chung Shan Medical University, No. 110, Sec. 1 Jianguo N. Rd, Taichung City, 40201 Taiwan; 3 School of Medicine, Chung Shan Medical University, Taichung City, Taiwan; 4 School of Medical Imaging and Radiological Sciences, Chung Shan Medical University, Taichung City, Taiwan; 5 School of Medical Informatics, Chung Shan Medical University, Taichung City, 40201 Taiwan; 6 Medical Imaging and Big Data Center, Chung Shan Medical University Hospital, Taichung City, Taiwan

**Keywords:** Smoking, PM_2.5_, cg05575921, Taiwan Biobank, Taiwan Air Quality Network

## Abstract

**Background:**

Polycyclic aromatic hydrocarbon (PAH)-rich substances like cigarette smoke and PM_2.5_ induce aryl hydrocarbon receptor (AHR)-mediated aryl hydrocarbon receptor repressor (AHRR) methylation. AHRR cg05575921 and coagulation factor II (thrombin) receptor-like 3 (F2RL3) cg03636183 methylation patterns are well-established biomarkers for smoking. Even though AHRR cg05575921 methylation has recently been associated with PM_2.5_, the interaction between smoking and PM_2.5_ on AHRR methylation is yet to be fully explored. We evaluated AHRR and F2RL3 CpG sites to identify potential significant markers in relation to PM_2.5_ and smoking in Taiwanese adults.

**Methods:**

DNA methylation and smoking data of 948 participants aged 30–70 years were obtained from the Taiwan Biobank Database (2008–2015), while PM_2.5_ data were obtained from the Air Quality Monitoring Database (2006–2011).

**Results:**

Smoking and PM_2.5_ were independently associated with hypomethylation (lower levels) of AHRR cg05575921, AHRR cg23576855, F2RL3 cg03636183, and F2LR3 cg21911711 after multiple-comparison correction (Bonferroni *P* < 0.00028409). Cg05575921 was the most hypomethylated AHRR CpG site, while cg03636183 was the most hypomethylated F2RL3 CpG site. Overall, cg05575921 was the most hypomethylated CpG site: *β* = − 0.03909, *P* < 0.0001; − 0.17536, *P* < 0.0001 for former and current smoking, respectively (P-trend_smoking_ < 0.0001) and − 0.00141, *P* < 0.0001 for PM_2.5_. After adjusting for F2RL3 cg03636183, smoking and PM_2.5_ remained significantly associated with cg05575921 hypomethylation: *β* − 0.02221, *P* < 0.0001; − 0.11578, *P* < 0.0001 for former and current smoking, respectively (P-trend_smoking_ < 0.0001) and − 0.0070, *P* = 0.0120 for PM_2.5_. After stratification by sex, smoking and PM_2.5_ remained associated (*P* < 0.05) with cg05575921 hypomethylation in both men (*β* = − 0.04274, − 0.17700, and − 0.00163 for former smoking, current smoking, and PM_2.5_, respectively) and women (*β* = − 0.01937, − 0.17255, and − 0.00105 for former smoking, current smoking, and PM_2.5_, respectively). After stratification by residential area, former and current smoking remained associated (*P* < 0.05) with cg05575921 hypomethylation: *β* = − 0.03918 and − 0.17536, respectively (P-trend_smoking_ < 0.0001). Living in the central and southern areas was also associated (*P* < 0.05) with cg05575921 hypomethylation: *β* = − 0.01356 and − 0.01970, respectively (P-trend_area_ < 0.0001).

**Conclusion:**

Smoking and PM_2.5_ were independently associated with hypomethylation of cg05575921, cg23576855, cg03636183, and cg21911711. The most hypomethylated CpG site was cg05575921 and its association with smoking and PM_2.5_ was dose-dependent.

## Background

Aryl hydrocarbon receptor plays a key role in the metabolism of xenobiotic substances like particulate matter and cigarette smoke which are rich in polycyclic aromatic hydrocarbons and dioxin [1–6]. This gene enhances tumor development through immunosuppression, proliferation, and apoptosis resistance [1, 7, 8]. The transcription factor, AHRR, is a tumor suppressor gene [1, 9, 10]. It modulates cancer enhancing processes like inflammation and proliferation [1, 11] by competing and repressing AHR. However, its downregulation causes an upsurge in AHR activities thereby enhancing tumorigenesis [10].

DNA methylation is a remarkable and stable covalent process that occurs during early tumorigenesis [12]. This epigenetic process is reversible and thus, is a potential diagnostic tool and therapeutic target for diseases especially lung cancer [12, 13]. Lung cancer is a major cause of cancer-related mortality in Taiwan [14, 15] and the world [13, 14, 16, 17]. Air pollution and cigarette smoking are two important environmental factors that independently enhance lung cancer development and mortality [16–24]. In 2015, approximately 8.9 and 6·4 million global deaths were attributable to PM_2.5_ and smoking, respectively [25, 26]. Significant joint effects of PM_2.5_ and smoking on lung cancer and cardiovascular disease mortality have been reported [27–29].

Hypomethylation of AHRR cg05575921 and F2RL3 cg03636183 was significantly associated with PAH exposure in a dose-dependent manner [30]. Moreover, these CpG sites have been consistently associated with smoking [17, 31–43] and lung cancer [10, 31, 35, 38, 42] and their methylation patterns are well-established biomarkers for smoking [41, 43]. AHRR is the most smoking-upregulated gene [44] and cg05575921 is the top-ranked smoking marker [45]. Even though cg05575921 methylation is markedly associated with smoking, its relationship with PM_2.5_ is yet to be fully established. To date, only one study has assessed the association between AHRR cg05575921 methylation and PM_2.5_ in Taiwan [46].

To our knowledge, no study has focused on the relationship of AHRR cg05575921 methylation with both PM_2.5_ and cigarette smoking. Hence, it is yet to be fully known whether PM_2.5_ and cigarette smoking have a joint effect on AHRR cg05575921 methylation. Therefore, the interaction between PM_2.5_ and smoking on AHRR cg05575921 methylation and other methylation markers for smoking merits more studies. In view of this, we evaluated AHRR and F2RL3 CpG sites to identify potential significant markers in relation to PM_2.5_ and smoking in Taiwanese men and women.

## Results

The association of AHRR and F2LR3 CpG sites with smoking and PM_2.5_ is shown in Supplementary Table 1. Four CpG sites (AHRR cg05575921, AHRR cg23576855, F2RL3 cg03636183, and F2RL3 cg21911711) had significant inverse associations with smoking and PM_2.5_ after multiple-comparison correction (Bonferroni *P* value < 0.00028409). That is, AHRR cg05575921, AHRR cg23576855, F2RL3 cg03636183, and F2RL3 cg21911711 were significantly hypomethylated in relation to smoking and PM_2.5_. Noteworthy, cg05575921 was the most hypomethylated AHRR CpG site, while cg03636183 was the most hypomethylated F2RL3 CpG site. In general, cg05575921 was the most hypomethylated CpG site (Supplementary Table 1) and further analysis was focused on this specific CpG site.

The clinical and demographic characteristics of 488 male and 460 female participants are illustrated in Table 1. The mean ± standard error (SE) AHRR cg05575921 methylation levels (beta-values) in men and women were 0.7832 ± 0.0042 and 0.8376 ± 0.0023, respectively. There were significant differences (*P <* 0.0001) between men and women with respect to cg05575921 methylation, smoking, alcohol drinking, and body mass index (BMI). The mean annual concentrations of PM_2.5_ from 2006 to 2011 were 26.53, 30.06, 36.91, and 40.68 μg/m^3^ for the northern, north-central, central, and southern area, respectively (Table 1).

**Table 1:** Basic characteristics of the study participants by sex

Variable	Men (*n* = 488)	Women (*n* = 460)	*P* value
AHRR cg05575921 (beta-value)	0.7832 ± 0.0042	0.8376 ± 0.0023	< 0.0001
F2RL3 cg03636183 (beta-value)	0.6334 ± 0.0025	0.6514 ± 0.0016	< 0.0001
Smoking status			< 0.0001
Never	279 (57.17%)	429 (93.26%)	
Former	117 (23.98%)	18 (3.91%)	
Current	92 (18.85%)	13 (2.83%)	
Area/PM_2.5_ levels (μg/m^3^)			0.7115
North/26.53	163 (33.40%)	167 (36.30%)	
North-center/30.06	81 (16.60%)	80 (17.39%)	
Center/36.91	102 (20.90%)	87 (18.91%)	
South/40.68	142 (29.10%)	126 (27.39%)	
Exercise			0.6106
No	271 (55.53%)	263 (57.17%)	
Yes	217 (44.47%)	197 (42.83%)	
Age (years)	50.1004 ± 0.5173	49.3391 ± 0.5108	0.2959
BMI (Kg/m^2^)	25.0637 ± 0.1507	23.5313 ± 0.1702	< 0.0001
Alcohol drinking			< 0.0001
No	396 (81.15%)	452 (98.26%)	
Former	32 (6.56%)	4 (0.87%)	
Current	60 (12.30%)	4 (0.87%)	
Secondhand smoke exposure			0.3259
No	426 (87.30%)	411 (89.35%)	
Yes	62 (12.70%)	49 (10.65%)	

Mean ± standard errors (SEs) represent continuous data and percentages (%) represent categorical data. The stated PM_2.5_ levels for each area are the mean concentrations from 2006–2011

Table 2 shows the association of smoking and PM_2.5_ with cg05575921 methylation in the study participants. Compared to never smoking, former and current smoking were significantly associated with lower levels of cg05575921: *β* = − 0.03909, *P* < 0.0001 and − 0.17536, *P* < 0.0001 for former and current smoking, respectively. Moreover, the test for linear trend was significant: P-trend_smoking_ < 0.0001 (Table 2). In addition to smoking, PM_2.5_, sex, and age were significantly associated with lower levels of cg05575921 methylation: *β* = − 0.00141 (*P* < 0.0001), − 0.01870 (*P* < 0.0001), and − 0.00044 (*P* = 0.0145) for PM_2.5_, male sex, and age, respectively (Table 2, model 1). Inclusion of F2RL3 cg03636183 into the model did not significantly affect the association of AHRR cg05575921 with smoking and PM_2.5_: *β* − 0.02221, *P* < 0.0001 and, − 0.11578, *P* < 0.0001 for former and current smoking, respectively (P-trend_smoking_ < 0.0001) and − 0.0070, *P* = 0.0120 for PM_2.5_ (Table 2, model 2). The interaction between PM_2.5_ and smoking on cg05575921 methylation was not significant.

**Table 2:** Multiple linear regression analysis showing the association of smoking and PM_2.5_ with AHRR cg05575921 methylation in the study participants

Variable	Model 1		Model 2	
	*β*	*P* value	*β*	*P* value
Smoking status (reference: never)				
Former	− 0.03909	< 0.0001	− 0.02221	< 0.0001
Current	− 0.17536	< 0.0001	− 0.11578	< 0.0001
P-trend	< 0.0001		< 0.0001	
PM_2.5_	− 0.00141	< 0.0001	− 0.00070	0.0120
Sex (reference: women)				
Men	− 0.01870	< 0.0001	− 0.01843	< 0.0001
Age	− 0.00044	0.0145	− 0.00018	0.2636
Exercise (reference: no)				
Yes	0.00077	0.8404	0.00052	0.8772
BMI	0.00060	0.2344	0.00014	0.7488
Alcohol drinking (reference: no)				
Former	− 0.00512	0.5931	− 0.00821	0.3264
Current	0.00157	0.8318	0.00539	0.4041
Secondhand smoke (reference: no)				
Yes	0.00136	0.8100	− 0.00204	0.6793
F2LR3 cg03636183 methylation	–	–	0.71185	< 0.0001

Model 1: not adjusted for F2LR3 cg03636183 methylation. Model 2: adjusted for F2LR3 cg03636183 methylation

After stratification by sex (Table 3), smoking and PM_2.5_ remained significantly associated with lower levels of cg05575921 methylation in both men: *β* = 0.04274, *P* < 0.0001 for former smoking; − 0.17700, *P* < 0.0001 for current smoking (P-trend_smoking_ < 0.0001); and − 0.00163, *P* = 0.0017 for PM_2.5_ and women: *β* = − 0.01937, *P* = 0.0417 for former smoking; − 0.17255, *P* < 0.0001 for current smoking (P-trend_smoking_ < 0.0001); and − 0.00105, *P* = 0.0015 for PM_2.5_ (Table 3).

**Table 3:** Multiple linear regression analysis showing the association of smoking and PM_2.5_ with AHRR cg05575921 methylation in the study participants stratified by sex

Variable	Men		Women	
	*β*	*P* value	β	*P* value
Smoking status (reference: never)				
Former	− 0.04274	< 0.0001	− 0.01937	0.0417
Current	− 0.17700	< 0.0001	− 0.17255	< 0.0001
P-trend	< 0.0001		< 0.0001	
PM_2.5_	− 0.00163	0.0017	− 0.00105	0.0015
Age	− 0.00045	0.1311	− 0.00033	0.0944
Exercise (reference: no)				
Yes	− 0.00015	0.9809	0.00292	0.4678
BMI	0.00001	0.9881	0.00103	0.0433
Alcohol drinking (reference: no)				
Former	− 0.01034	0.4041	0.03269	0.1040
Current	0.00127	0.8913	0.03218	0.1065
Secondhand smoke exposure (reference: no)				
Yes	− 0.00070	0.9394	0.00366	0.5525

Table 4 shows the association of smoking and living in PM_2.5_ areas with cg05575921 methylation. With never smoking as the reference variable, significant lower levels of AHRR cg05575921 methylation were observed in both former and current smokers: *β* = − 0.03918, *P* < 0.0001 and − 0.17536, *P* < 0.0001, respectively (P-trend_smoking_ < 0.0001). Living in areas with higher PM_2.5_ pollution was associated with lower levels of cg05575921 methylation: *β* = − 0.00267, *P =* 0.6230 for the north-central; − 0.01356, *P* = 0.0074 for the central; and − 0.01970, *P* < 0.0001 for the southern area. Despite the absence of an association between living in the north-central area and cg05575921 methylation, the test for trend was significant (P-trend_area_ < 0.0001).

**Table 4:** Multiple linear regression analysis showing the association of smoking and living in PM_2.5_ areas with AHRR (cg05575921) methylation in the study participants

Variable	*β*	*P* value
Smoking status (reference: never)		
Former	− 0.03918	< 0.0001
Current	− 0.17536	< 0.0001
P-trend	< 0.0001	
Area (reference: north)		
North-center	− 0.00267	0.6230
Center	− 0.01356	0.0074
South	− 0.01970	< 0.0001
P-trend	< 0.0001	
Exercise (reference: no)		
Yes	0.00086	0.8218
Sex (reference: women)		
Men	− 0.01867	< 0.0001
Age	− 0.00044	0.0139
BMI	0.00058	0.2527
Alcohol drinking (reference: no)		
Former	− 0.00500	0.6027
Current	0.00156	0.8330
Secondhand smoke exposure (reference: no)		
Yes	0.00139	0.8063

After stratification by sex (Table 5), smoking and living in areas with higher PM_2.5_ pollution remained significantly associated with lower levels of AHRR cg05575921 methylation in both men: *β* = − 0.01996, *P* = 0.0374 for former smoking; − 0.17221, *P* < 0.0001 for current smoking (P-trend_smoking_ < 0.0001); − 0.00577, *P* = 0.3174 for the north-central; − 0.00935, *P* = 0.0820 for the central; and − 0.01620, *P* = 0.0016 for the southern area (P-trend_area_ = 0.0013) and women: *β* = − 0.04276, *P* < 0.0001 for former smoking; − 0.17706, *P* < 0.0001 for current smoking (P-trend_smoking_ < 0.0001); and 0.00092, *P* = 0.9198 for the north-central; − 0.01770, *P* = 0.0361 for the central; and − 0.02076, *P* = 0.0092 for the southern area (P-trend_area_ = 0.0025).

**Table 5:** Multiple linear regression analysis showing the association of smoking and living in PM_2.5_ areas with AHRR (cg05575921) methylation in the study participants stratified by sex

Variable	Men		Women	
	*β*	*P* value	*β*	*P* value
Smoking status (reference: never)				
Former	− 0.01996	0.0374	− 0.04276	< 0.0001
Current	− 0.17221	< 0.0001	− 0.17706	< 0.0001
P-trend	< 0.0001		< 0.0001	
Area (reference: north)				
North-center	− 0.00577	0.3174	0.00092	0.9198
Center	− 0.00935	0.0820	− 0.01770	0.0361
South	− 0.01620	0.0016	− 0.02076	0.0092
P-trend	0.0013		0.0025	
Exercise (no)				
Yes	0.00294	0.4651	0.00001	0.9986
Age	− 0.00033	0.0939	− 0.00045	0.1278
BMI	0.00105	0.0407	− 0.00006	0.9477
Alcohol drinking (reference: no)				
Former	0.03356	0.0977	− 0.00934	0.4539
Current	0.03177	0.1127	0.00115	0.9022
Secondhand smoke exposure (reference: no)				
Yes	0.00361	0.5589	− 0.00060	0.9489

The association of F2RL3 cg03636183 (another smoking-related CpG site) with PM_2.5_ and smoking was similar to that observed for AHRR cg05575921. That is, PM_2.5_ and smoking were significantly associated with hypomethylation of F2RL3 cg03636183 (Supplementary Tables 2, 3, 4 and 5).

## Discussion

In the current study, smoking and PM_2.5_ were significantly associated with hypomethylation of AHRR cg05575921, AHRR cg23576855, F2RL3 cg03636183, and F2LR3 cg21911711 after multiple-comparison correction (Bonferroni correction). Cg05575921 was the most hypomethylated AHRR CpG site, while cg03636183 was the most hypomethylated F2RL3 CpG site. Noteworthy, cg05575921 was the overall top significant CpG site and its relation with smoking and PM_2.5_ was dose-dependent. Adjusting for F2RL3 cg03636183 did not affect the association of smoking and PM_2.5_ with cg05575921. However, there was no significant interaction between smoking and PM_2.5_ on cg05575921 methylation. To the best of our knowledge, no study has assessed both the independent and joint association of smoking and PM_2.5_ with AHRR cg05575921 methylation.

Cigarette smoke contains toxic components capable of directly altering the epigenetic profiles of white blood cells in the bloodstream [39]. For instance, PAH in cigarette smoke induces AHR-mediated AHRR expression and methylation [1, 2, 5, 47, 48]. In light of this, we focused on the association of smoking and PM_2.5_ with a specific AHRR methylation marker, cg05575921. Our focus on this particular CpG site was because it has recently been associated with PM_2.5_ exposure in non-smokers [46]. Besides, it has consistently shown dose-response relationships with smoking [17, 31–40, 49] and has also shown high sensitivity and specificity in predicting smoking status [33, 50, 51]. Moreover, it has been the most significant hypomethylated smoking-related CpG site in many epigenome-wide association studies (EWAS) [45]. Findings from the current study are in concordance with those stated above.

In addition to being a predictor of smoking habits [52], AHRR cg05575921 methylation from whole blood or saliva DNA is a validated predictor of smoking-related morbidity and mortality [35, 49]. For instance, AHRR cg05575921 hypomethylation has been associated with enhanced carcinogenesis especially in the lungs [10, 31, 35, 38]. The mechanism underlying the relationship between AHRR cg05575921 methylation and lung cancer is unclear. However, cigarette smoke-induced AHRR hypomethylation and the associated increase in the expression of the AHRR gene could compromise the metabolizing ability of the body. This impairs the removal of deleterious environmental pollutants thereby increasing the risk of cancer [53]. Moreover, it is believed that cigarette smoke triggers vascular endothelial dysfunction by disrupting the structure of the airway epithelial barrier [54, 55]. This eases the entry, deposition, and retention of particles into the arterial wall [56, 57]. Cigarette smoke may also impair the clearance of fine particles in the alveoli thereby aggravating subsequent deleterious effects [56, 57].

Toxic components adsorbed on the surface of PM_2.5_ like PAHs have been associated with DNA methylation changes [58, 59]. Like PM_2.5_, cigarette smoke also contains PAHs which are capable of inducing AHR-mediated AHRR methylation [1, 2, 5, 47, 48]. Since both cigarette smoke and PM_2.5_ are inhalable carcinogens rich in PAH, PM_2.5_-induced AHRR methylation might also explain the pathophysiological mechanism of lung cancer. While the relationship of AHRR cg05575921 methylation with smoking is well-documented [17, 31–40, 49], PM_2.5_-related AHRR cg05575921 methylation on the other hand still deserves more attention. It has been suggested that air pollution could be the driving force behind the AHRR cg05575921 hypomethylation-associated impaired lung function in never smokers [60]. In our previous study, PM_2.5_ was significantly associated with AHRR cg05575921 hypomethylation in non-smoking Taiwanese adults [46]. In the current study, PM_2.5_ was also inversely associated with AHRR cg05575921 methylation in both smoking and non-smoking Taiwanese adults.

In the current study, PM_2.5_ and smoking were also significantly associated with hypomethylation of F2RL3 cg03636183, another prominent smoking-related methylation site. The smoking-related F2RL3 cg03636183 hypomethylation observed in this study is in line with findings from previous studies [31, 41, 42, 45, 61–63]. Note should be taken that AHRR cg05575921 remained the top significant hypomethylated CpG site in the current study. Moreover, its association with smoking and PM_2.5_ remained significant even after adjusting for F2RL3 cg03636183.

Significant interactions between smoking and PM_2.5_ on lung cancer and cardiovascular mortality [27–29] have been reported. However, we found no significant interactions between smoking and PM_2.5_ on cg05575921 AHRR methylation. The smoking-related cg05575921 methylation levels were lower compared to the PM_2.5_-related levels. Despite this, the association of PM_2.5_ with cg05575921 methylation cannot be neglected because exposure to air pollution is involuntary and affects everyone, unlike smoking which is individual behavior that can be modified [64]. To our knowledge, there are no available publications on the interaction of PM_2.5_ with smoking on cg05575921 methylation. In view of this, we recommend further research in this area.

The relatively large sample size in addition to the adjusting for secondhand smoke exposure constitute the strengths of this study. However, the study is limited in that there was no available data to evaluate the functional correlation between mRNA gene expression and AHRR cg05575921 methylation. Moreover, all eligible Taiwan Biobank participants are individuals who have no personal history of cancer. Therefore, our results may not truly depict the pattern of cg05575921 methylation in individuals with cancer. Furthermore, since validated tools for individual exposure estimates are not available, the actual concentrations of PM_2.5_ in individuals could not be determined. The use of nearby monitoring stations to estimate exposure to PM_2.5_ might have resulted in exposure classification error. Smoking was self-reported and the possibility of recall bias cannot be ruled out. However, our findings were consistent with several previous findings on smoking and cg05575921 methylation [17, 31–40, 49].

## Conclusion

Smoking and PM_2.5_ were independently associated with lower levels of AHRR cg05575921, AHRR cg23576855, F2RL3 cg03636183, and F2RL3 cg21911711 after multiple-comparison correction. The most hypomethylated CpG site was cg05575921 and its association with smoking and PM_2.5_ was in a dose-dependent manner. Even though both smoking and PM_2.5_ were inversely associated with cg05575921 methylation, the smoking-related methylation levels were relatively low. Despite this, PM_2.5_-related methylation cannot be ignored because exposure to air pollution is involuntary and could affect everyone while smoking is a modifiable individual behavior. Our findings suggest that smoking and PM_2.5_ may independently but not jointly affect AHRR cg05575921 methylation. Considering the remarkable harmful impact of PM_2.5_, smoking, and cg05575921 hypomethylation on cardiovascular and pulmonary health, measures should be put in place to reduce PM_2.5_ pollution and individuals should be sensitized on the harmful effects of smoking.

## Methods

### Study participants and data sources

A total of 948 individuals (488 men and 460 women) were included in the current study. Genomic and demographic data including DNA methylation, smoking, residence, exercise, age, BMI, alcohol drinking, and secondhand smoke were obtained from the Taiwan Biobank Database (2008–2015), while PM_2.5_ data were obtained from the Air Quality Monitoring Database (2006–2011).

The Taiwan Biobank project is a community-based cohort study aimed at collecting genetic and lifestyle data, alongside tracking the health of more than 200,000 ethnic Taiwanese adults for at least 10 years [65, 66]. This national health resource contains 29 recruitment centers that are distributed all over the island [65]. Only Taiwanese adults aged 30–70 years who have no personal history of cancer are eligible for recruitment [65]. Relevant guidelines and regulations are followed during the recruitment process. All eligible participants signed a letter of consent before the data collection process.

Long-term and well-maintained air quality monitoring systems are a major basis of effective air quality protection and control. Air quality monitoring in Taiwan began in 1993 and is run by the Taiwan Air Quality Monitoring Network (TAQMN) of the Environmental Protection Administration (EPA) [67, 68]. More information about this TAQMN has been described elsewhere [69]. Currently, there are about 77 fully automated air quality monitoring stations distributed throughout Taiwan which provide daily levels of air pollutants [68, 70]. The TAQMN began providing regular daily mean measures of PM_2.5_ since 2006 [70].

### DNA Methylation determination

DNA methylation experiments and analyses were performed by the Health GeneTech Corporation in Taiwan. In brief, sodium bisulfite treatment of DNA extracted from whole blood was done using the EZ DNA Methylation Kit (Zymo Research, CA, USA). Methylation levels were measured using the Illumina Infinium MethylationEPIC BeadChip (Illumina Inc., San Diego, CA, USA) which detects over 850,000 CpG sites within the human genome [71, 72]. Methylation levels were represented by *β* values which range from 0 (no methylation) to 1 (full methylation). To ensure good quality control, probes with poor detection (*P* > 0.05) and bead count < 3 were excluded, while background signals were subtracted. In addition, dye-bias across batches was corrected by normalization and outliers were removed using the median absolute deviation method. Finally, cell-type heterogeneity was corrected using the Reference-Free Adjustment for Cell-Type composition (ReFACTor) method [73].

### Exposure and covariate assessment

We estimated exposure to PM_2.5_ using participants’ residential addresses which were grouped into 4 (northern, north-central, central, and southern area) as previously described [22, 46]. Annual average concentrations of PM_2.5_ from 2006 to 2011 were used in the final analysis. Smoking habits were self-reported and participants were classified as current, former, or non-smokers. Covariates included exercise, sex, age, BMI, alcohol drinking habits (current, former and non-drinking), and secondhand smoke exposure. Smoking habits and covariates were defined using the same criteria described by Tantoh and colleagues [46].

### Statistical analysis

Data management and statistical analyses were performed using the SAS 9.3 software (SAS Institute, Cary, NC). Continuous data were analyzed using the *t* test and expressed as mean ± standard error (SE), while categorical data were analyzed using the chi-square test and expressed as percentages (%). Multivariate linear regression models were used to determine the association of smoking and PM_2.5_ with AHRR and F2RL3 CpG sites. The most significant Bonferroni-corrected CpG site was used for further analysis. Adjustments were made for covariates including exercise, sex, age, BMI, alcohol drinking, and secondhand smoke exposure.

## Supplementary information

**Additional file 1: Table S1.** AHRR and F2LR3 CpG sites and their association with smoking and PM_2.5_. **Table S2.** Multiple linear regression analysis showing the association of smoking and PM_2.5_ with F2RL3 cg03636183 methylation in the study participants. **Table S3.** Multiple linear regression analysis showing the association of smoking and PM_2.5_ with F2RL3 cg03636183 methylation in the study participants stratified by sex. **Table S4.** Multiple linear regression analysis showing the association of smoking and living in PM_2.5_ areas with F2RL3 cg03636183 methylation in the study participants. **Table S5.** Multiple linear regression analysis showing the association of smoking and living in PM_2.5_ areas and F2RL3 cg03636183 methylation in the study participants stratified by sex.

## Data Availability

The data that support the findings of this study are available from Taiwan Biobank but restrictions apply to the availability of these data, which were used under license for the current study, and so are not publicly available. Data are however available from the authors upon reasonable request and with permission of Taiwan Biobank.

## Abbreviations

PAH: Polycyclic aromatic hydrocarbon
PM_2.5_: Particulate matter < 2.5 microns in aerodynamic diameter
AHR: Aryl hydrocarbon receptor
AHRR: Aryl hydrocarbon receptor repressor
F2RL3: Coagulation factor II (thrombin) receptor-like 3
DNA: Deoxyribonucleic acid
SE: Standard error
*β*: Regression coefficient
TAQMN: Taiwan Air Quality Monitoring Network
EPA: Environmental Protection Administration
CpG: Cytosine-phosphate-guanine
BMI: Body mass index
MOST: Ministry of Science and Technology
*n*: Sample size
