# Supplementary material for: AHRR cg05575921 methylation in relation to smoking and PM2.5 exposure among Taiwanese men and women
Source: Clin Epigenetics. 2020 Jul 31;12:117. doi: 10.1186/s13148-020-00908-3 (PMC7394684; doi:10.1186/s13148-020-00908-3)
Supplement: Supplementary file 1 — Additional file 1: Table S1. AHRR and F2LR3 CpG sites and their association with smoking and PM2.5. Table S2. Multiple linear regression analysis showing the association of smoking and PM2.5 with F2RL3 cg03636183 methylation in the study participants. Table S3. Multiple linear regression analysis showing the association of smoking and PM2.5 with F2RL3 cg03636183 methylation in the study participants stratified by sex. Table S4. Multiple linear regression analysis showing the association of smoking and living in PM2.5 areas with F2RL3 cg03636183 methylation in the study participants. Table S5. Multiple linear regression analysis showing the association of smoking and living in PM2.5 areas and F2RL3 cg03636183 methylation in the study participants stratified by sex. [file 13148_2020_908_MOESM1_ESM.docx]

Supplementary Table 1. AHRR and F2LR3 CpG sites and their association with smoking and PM_2.5_.

| Gene | CpG site | Smoking status | | | | PM_2.5_ | |
| --- | --- | --- | --- | --- | --- | --- | --- |
|  |  | Former | | Current | |  |  |
|  |  | β | P-value | β | P-value | β | P-value |
| **AHRR** | **cg05575921** | **-0.03909** | **<0.00001**** | **-0.17536** | **<0.00001**** | **-0.00141** | **0.00001**** |
| **AHRR** | **cg23576855** | **-0.03519** | **<0.00001**** | **-0.14079** | **<0.00001**** | **-0.00139** | **0.00005**** |
| AHRR | cg05934812 | -0.00676 | 0.0043* | -0.01959 | 0.00000** | -0.00067 | 0.00000** |
| AHRR | cg12806681 | -0.00602 | 0.00534* | -0.02045 | 0.00000** | -0.00056 | 0.00000** |
| AHRR | cg26703534 | -0.00873 | 0.00347* | -0.03662 | 0.00000** | -0.00144 | 0.00000** |
| AHRR | cg04551776 | -0.00778 | 0.01436* | -0.02293 | 0.00000** | -0.00114 | 0.00000** |
| AHRR | cg25648203 | -0.00668 | 0.01763* | -0.03415 | 0.00000** | -0.00081 | 0.00000** |
| AHRR | cg21161138 | -0.00944 | 0.00042* | -0.04874 | 0.00000** | -0.00074 | 0.00000** |
| AHRR | cg11258653 | -0.00307 | 0.04661* | -0.00383 | 0.02862* | -0.00042 | 0.00000** |
| AHRR | cg04135110 | 0.00873 | 0.0251* | 0.01682 | 0.00014** | 0.00048 | 0.02944* |
| AHRR | cg17287155 | -0.00475 | 0.07214 | -0.01885 | 0.00000** | -0.00114 | 0.00000** |
| AHRR | cg21813876 | -0.00171 | 0.13265 | -0.00304 | 0.01802* | -0.00033 | 0.00000** |
| AHRR | cg09854184 | -0.00231 | 0.17592 | -0.00415 | 0.03206* | -0.00057 | 0.00000** |
| AHRR | cg09634134 | 0.00136 | 0.60803 | 0.00713 | 0.01757* | 0.00086 | 0.00000** |
| AHRR | cg08606254 | 0.00229 | 0.26431 | 0.00588 | 0.01151* | -0.00065 | 0.00000** |
| AHRR | cg24980413 | 0.00231 | 0.63804 | 0.013 | 0.01964* | -0.00108 | 0.00011** |
| AHRR | cg18615970 | -0.00732 | 0.09525 | -0.01421 | 0.00423* | -0.00135 | 0.00000** |
| AHRR | cg16219322 | -0.00154 | 0.39454 | -0.0072 | 0.00047* | -0.00066 | 0.00000** |
| AHRR | cg11557553 | -0.00015 | 0.93575 | 0.00619 | 0.00348* | -0.00063 | 0.00000** |
| AHRR | cg26529655 | -0.00232 | 0.42573 | -0.00661 | 0.04551* | -0.00125 | 0.00000** |
| AHRR | cg26850624 | 0.00236 | 0.34601 | 0.00813 | 0.00426* | -0.00075 | 0.00000** |
| AHRR | cg03569073 | -0.00508 | 0.00046* | -0.00131 | 0.42371 | -0.0003 | 0.00021** |
| AHRR | cg17166056 | -0.00393 | 0.01171* | 0.00023 | 0.89732 | -0.00043 | 0.00000** |
| AHRR | cg18205372 | -0.00495 | 0.02147* | 0.0013 | 0.59532 | -0.00075 | 0.00000** |
| AHRR | cg14219121 | 0.00057 | 0.73935 | 0.00731 | 0.00019** | -0.00026 | 0.00744* |
| AHRR | cg11902777 | -0.00414 | 0.11851 | -0.01702 | 0.00000** | 0.00036 | 0.01524* |
| AHRR | cg21144161 | -0.00128 | 0.49373 | 0.00829 | 0.00009** | -0.00024 | 0.02314* |
| AHRR | cg04369835 | 0.01801 | 0.02295* | 0.02597 | 0.00398* | 0.00116 | 0.01006* |
| AHRR | cg11763982 | -0.00619 | 0.00718* | -0.00784 | 0.00265* | -0.0004 | 0.00208* |
| AHRR | cg10841124 | 0.00979 | 0.00282* | 0.0093 | 0.01194* | 0.00047 | 0.01031* |
| AHRR | cg00731338 | 0.01298 | 0.01814* | 0.03675 | 0.00000** | -0.00042 | 0.17548 |
| AHRR | cg07943658 | 0.0092 | 0.00328* | 0.02329 | 0.00000** | 0.00031 | 0.07496 |
| AHRR | cg23916896 | -0.01168 | 0.0325* | -0.03703 | 0.00000** | 0.00039 | 0.19989 |
| AHRR | cg01899089 | -0.0126 | 0.00442* | -0.02563 | 0.00000** | -0.00002 | 0.94355 |
| AHRR | cg11554391 | -0.00524 | 0.02927* | -0.01203 | 0.00001** | 0.0002 | 0.13298 |
| AHRR | cg26987759 | 0.00967 | 0.04088* | 0.02101 | 0.00009** | 0.00024 | 0.36672 |
| AHRR | cg07780979 | -0.00294 | 0.08136 | 0.00015 | 0.93745 | -0.00065 | 0.00000** |
| AHRR | cg27240182 | -0.00202 | 0.17493 | 0.0007 | 0.68098 | -0.0004 | 0.00000** |
| AHRR | cg06047773 | -0.00105 | 0.6945 | 0.00014 | 0.96268 | -0.00091 | 0.00000** |
| AHRR | cg04939692 | -0.00148 | 0.3156 | 0.00069 | 0.6796 | -0.00043 | 0.00000** |
| AHRR | cg09662430 | -0.00008 | 0.96862 | 0.00055 | 0.80821 | 0.0007 | 0.00000** |
| AHRR | cg06605558 | -0.00054 | 0.70493 | -0.00118 | 0.46953 | -0.00039 | 0.00000** |
| AHRR | cg17310215 | -0.00039 | 0.87655 | -0.00241 | 0.39402 | -0.00054 | 0.00016** |
| AHRR | cg13707777 | 0.00051 | 0.90972 | -0.00418 | 0.41446 | -0.00157 | 0.00000** |
| AHRR | cg14982043 | 0.00067 | 0.73827 | -0.00046 | 0.83951 | -0.00056 | 0.00000** |
| AHRR | cg13404472 | -0.00209 | 0.25343 | 0.00001 | 0.99764 | -0.00045 | 0.00002** |
| AHRR | cg09470163 | 0.00204 | 0.17172 | 0.00163 | 0.33542 | 0.00068 | 0.00000** |
| AHRR | cg07137034 | 0.0026 | 0.57619 | 0.00256 | 0.62727 | -0.00158 | 0.00000** |
| AHRR | cg01970407 | 0.00077 | 0.80457 | -0.00042 | 0.90452 | -0.00083 | 0.00000** |
| AHRR | cg05758931 | -0.0045 | 0.30272 | -0.00688 | 0.16371 | 0.00091 | 0.00025** |
| AHRR | cg09078014 | -0.00611 | 0.06123 | -0.00629 | 0.08856 | -0.00142 | 0.00000** |
| AHRR | cg24688690 | 0.0008 | 0.71755 | -0.00213 | 0.39205 | -0.00065 | 0.00000** |
| AHRR | cg05655106 | -0.001 | 0.55464 | -0.00103 | 0.59009 | -0.00069 | 0.00000** |
| AHRR | cg26487191 | 0.00223 | 0.38697 | 0.00036 | 0.90067 | 0.00073 | 0.00000** |
| AHRR | cg00629928 | -0.00207 | 0.13536 | -0.0003 | 0.85044 | -0.00056 | 0.00000** |
| AHRR | cg04202140 | -0.00482 | 0.1059 | -0.00496 | 0.14134 | -0.00173 | 0.00000** |
| AHRR | cg08714121 | 0.00135 | 0.37835 | 0.00325 | 0.06194 | 0.00036 | 0.00003** |
| AHRR | cg22356527 | 0.00115 | 0.54552 | 0.00312 | 0.14798 | 0.00065 | 0.00000** |
| AHRR | cg17472786 | -0.00369 | 0.09428 | -0.00281 | 0.25903 | -0.00047 | 0.00017** |
| AHRR | cg04066994 | -0.00128 | 0.5217 | 0.00104 | 0.64697 | -0.00042 | 0.00017** |
| AHRR | cg12937950 | -0.00451 | 0.37184 | -0.00539 | 0.3422 | -0.00131 | 0.00000** |
| AHRR | cg22698028 | 0.00039 | 0.86912 | -0.00163 | 0.545 | 0.00059 | 0.00001** |
| AHRR | cg19405895 | -0.00171 | 0.15713 | -0.0016 | 0.23961 | -0.00034 | 0.00000** |
| AHRR | cg21880882 | -0.00376 | 0.10128 | -0.00042 | 0.87279 | -0.00054 | 0.00003** |
| AHRR | cg09078081 | -0.00074 | 0.58829 | -0.00037 | 0.8135 | -0.0003 | 0.00011** |
| AHRR | cg14684960 | -0.00349 | 0.07649 | 0.00201 | 0.3672 | -0.00055 | 0.00000** |
| AHRR | cg09584122 | -0.00092 | 0.47997 | -0.00038 | 0.79815 | -0.00051 | 0.00000** |
| AHRR | cg11610050 | 0.00029 | 0.84807 | -0.00146 | 0.40257 | -0.00062 | 0.00000** |
| AHRR | cg16049691 | -0.00123 | 0.56138 | 0.0034 | 0.15599 | -0.00072 | 0.00000** |
| AHRR | cg16336872 | -0.00214 | 0.33868 | -0.00086 | 0.73533 | -0.00103 | 0.00000** |
| AHRR | cg14807090 | 0.00069 | 0.7323 | 0.00328 | 0.15355 | -0.00053 | 0.00000** |
| AHRR | cg26954197 | 0.00209 | 0.31562 | 0.0041 | 0.08266 | 0.0006 | 0.00000** |
| AHRR | cg05842815 | 0.00042 | 0.80331 | -0.00255 | 0.18316 | -0.0005 | 0.00000** |
| AHRR | cg06035956 | -0.00248 | 0.08344 | -0.00854 | 0.00000** | -0.0001 | 0.22658 |
| AHRR | cg24090911 | -0.00254 | 0.39785 | -0.02159 | 0.00000** | -0.00031 | 0.06821 |
| AHRR | cg08902828 | -0.00258 | 0.38462 | -0.01982 | 0.00000** | -0.00011 | 0.50556 |
| AHRR | cg22030839 | -0.00222 | 0.04413* | -0.00154 | 0.21731 | -0.00022 | 0.0005* |
| AHRR | cg00300637 | 0.00588 | 0.05012 | 0.00856 | 0.01179* | -0.00043 | 0.01113* |
| AHRR | cg04141806 | -0.00061 | 0.73932 | -0.00512 | 0.01386* | 0.00021 | 0.04186* |
| AHRR | cg22937882 | 0.00272 | 0.1304 | 0.00593 | 0.0036* | -0.00035 | 0.00069* |
| AHRR | cg00699559 | 0.00448 | 0.07633 | 0.00667 | 0.01994* | 0.00042 | 0.00312* |
| AHRR | cg08519949 | -0.00027 | 0.79273 | -0.00082 | 0.47898 | 0.00016 | 0.00662* |
| AHRR | cg12845747 | 0.00021 | 0.91036 | -0.00096 | 0.64726 | -0.00035 | 0.0008* |
| AHRR | cg11148817 | -0.00115 | 0.37956 | -0.00216 | 0.14591 | -0.00022 | 0.00245* |
| AHRR | cg14453201 | -0.00222 | 0.41217 | -0.00303 | 0.32548 | 0.00045 | 0.00318* |
| AHRR | cg06802630 | 0.01294 | 0.07023 | 0.01241 | 0.12574 | 0.00086 | 0.03335* |
| AHRR | cg02356223 | -0.0009 | 0.5355 | -0.002 | 0.22461 | -0.00023 | 0.00553* |
| AHRR | cg09084391 | -0.00195 | 0.45007 | -0.00155 | 0.5965 | -0.00038 | 0.01029* |
| AHRR | cg02088390 | 0.00027 | 0.87038 | 0.00118 | 0.53258 | -0.00019 | 0.04557* |
| AHRR | cg24130459 | -0.00007 | 0.96774 | -0.00077 | 0.68761 | -0.00026 | 0.00757* |
| AHRR | cg24256039 | 0.00322 | 0.56186 | 0.00033 | 0.95786 | 0.00081 | 0.00992* |
| AHRR | cg11894422 | -0.00151 | 0.45466 | 0.00042 | 0.85282 | -0.00025 | 0.02638* |
| AHRR | cg03604011 | 0.00034 | 0.86326 | 0.00217 | 0.33006 | -0.00033 | 0.00281* |
| AHRR | cg05527650 | 0.00374 | 0.27777 | -0.00294 | 0.45147 | 0.00066 | 0.00072* |
| AHRR | cg05597431 | 0.00168 | 0.53157 | 0.00435 | 0.15337 | -0.00049 | 0.0012* |
| AHRR | cg09478603 | -0.00017 | 0.97709 | 0.00651 | 0.32881 | -0.00072 | 0.02876* |
| AHRR | cg00976097 | -0.00019 | 0.96754 | 0.00728 | 0.16839 | -0.00094 | 0.00038* |
| AHRR | cg22816059 | 0.00021 | 0.88028 | 0.00186 | 0.24788 | -0.00017 | 0.03626* |
| AHRR | cg16189952 | -0.00177 | 0.62659 | 0.00265 | 0.5201 | -0.00055 | 0.00744* |
| AHRR | cg11445754 | -0.00079 | 0.57989 | 0.00237 | 0.14246 | -0.00029 | 0.00041* |
| AHRR | cg07448928 | 0.00009 | 0.97316 | -0.00425 | 0.15622 | -0.00053 | 0.00045* |
| AHRR | cg16577724 | -0.00049 | 0.68518 | -0.00011 | 0.93607 | 0.00022 | 0.00112* |
| AHRR | cg16172278 | 0.0007 | 0.67589 | 0.00184 | 0.34126 | -0.00021 | 0.03085* |
| AHRR | cg02385153 | 0.00575 | 0.11311 | 0.00752 | 0.06812 | 0.00064 | 0.00191* |
| AHRR | cg22103736 | -0.0029 | 0.02296* | -0.00187 | 0.19838 | 0.00002 | 0.77053 |
| AHRR | cg06678548 | -0.00668 | 0.01259* | -0.00389 | 0.19911 | -0.00017 | 0.2509 |
| AHRR | cg20433154 | 0.00618 | 0.00517* | 0.00317 | 0.20507 | -0.00015 | 0.22397 |
| AHRR | cg14690983 | 0.00403 | 0.02713* | 0.00245 | 0.23495 | 0.00014 | 0.1807 |
| AHRR | cg17386114 | -0.0028 | 0.04492* | 0.00082 | 0.6049 | -0.00004 | 0.57387 |
| AHRR | cg01097768 | -0.00146 | 0.4966 | -0.00869 | 0.00037* | -0.00012 | 0.30866 |
| AHRR | cg09338136 | -0.00145 | 0.61251 | -0.00917 | 0.00474* | 0.00011 | 0.50074 |
| AHRR | cg16501378 | -0.00115 | 0.43788 | 0.00427 | 0.01076* | 0.00012 | 0.15726 |
| AHRR | cg24891125 | -0.0029 | 0.1698 | 0.00609 | 0.011* | -0.00014 | 0.25557 |
| AHRR | cg14454127 | -0.00118 | 0.52481 | -0.00509 | 0.01518* | -0.00018 | 0.08289 |
| AHRR | cg14448919 | -0.00015 | 0.93705 | 0.00525 | 0.01806* | -0.00011 | 0.30972 |
| AHRR | cg01141993 | -0.00049 | 0.73663 | 0.0037 | 0.02607* | -0.00014 | 0.08295 |
| AHRR | cg18584368 | -0.00021 | 0.87545 | -0.00319 | 0.03227* | -0.00008 | 0.29767 |
| AHRR | cg05516328 | -0.00981 | 0.2488 | -0.02059 | 0.03266* | -0.00017 | 0.71633 |
| AHRR | cg03561637 | -0.0004 | 0.8264 | -0.00431 | 0.03622* | 0.00004 | 0.6878 |
| AHRR | cg12202185 | -0.00784 | 0.0529 | -0.00943 | 0.0399* | 0.0003 | 0.18955 |
| AHRR | cg10869925 | 0.00064 | 0.70174 | 0.00386 | 0.04264* | 0.00008 | 0.40242 |
| AHRR | cg17989581 | -0.00068 | 0.39774 | -0.00176 | 0.04874* | -0.00006 | 0.21667 |
| AHRR | cg21972741 | -0.00825 | 0.00667* | -0.00046 | 0.89377 | -0.00031 | 0.07254 |
| AHRR | cg12207033 | -0.0005 | 0.78127 | 0.00395 | 0.05417 | -0.00016 | 0.12275 |
| AHRR | cg15168497 | 0.00446 | 0.44277 | -0.0126 | 0.05667 | -0.00043 | 0.19224 |
| AHRR | cg19442702 | -0.00083 | 0.59575 | 0.0034 | 0.05756 | 0.00012 | 0.1808 |
| AHRR | cg09454315 | 0.00956 | 0.20062 | 0.0156 | 0.06623 | 0.00041 | 0.33087 |
| AHRR | cg26076054 | 0.00082 | 0.93237 | 0.01957 | 0.07403 | -0.00097 | 0.07508 |
| AHRR | cg13023972 | -0.00475 | 0.09062 | -0.00557 | 0.08022 | 0.00003 | 0.85144 |
| AHRR | cg04021706 | 0.00175 | 0.35822 | 0.00374 | 0.08252 | -0.00006 | 0.57343 |
| AHRR | cg21623028 | 0.00198 | 0.44481 | 0.00468 | 0.11111 | -0.00022 | 0.13062 |
| AHRR | cg08491376 | 0.00004 | 0.97738 | 0.00259 | 0.13488 | -0.00016 | 0.07317 |
| AHRR | cg14647125 | 0.00469 | 0.24813 | -0.00673 | 0.14031 | -0.00017 | 0.45098 |
| AHRR | cg16294152 | -0.00126 | 0.72986 | -0.00606 | 0.14303 | -0.00008 | 0.71494 |
| AHRR | cg08802770 | -0.00089 | 0.17683 | -0.00101 | 0.18392 | -0.00003 | 0.36948 |
| AHRR | cg20344367 | 0.00189 | 0.25504 | 0.00237 | 0.20724 | 0.00011 | 0.24704 |
| AHRR | cg03491025 | -0.00181 | 0.28312 | -0.00236 | 0.21778 | -0.00015 | 0.11247 |
| AHRR | cg03891523 | -0.0012 | 0.56646 | -0.00289 | 0.22128 | -0.00021 | 0.07857 |
| AHRR | cg17248487 | 0.00239 | 0.31572 | -0.00304 | 0.2589 | -0.00007 | 0.59453 |
| AHRR | cg24081180 | -0.00077 | 0.67338 | 0.00233 | 0.25949 | -0.00001 | 0.88622 |
| AHRR | cg26320890 | -0.00103 | 0.73407 | -0.00387 | 0.26059 | -0.00012 | 0.50082 |
| AHRR | cg01958142 | 0.00362 | 0.09464 | -0.00272 | 0.26785 | 0.00017 | 0.17379 |
| AHRR | cg06036945 | -0.00018 | 0.80302 | 0.00084 | 0.31902 | 0.00001 | 0.82889 |
| AHRR | cg05601199 | 0.00067 | 0.3371 | 0.00075 | 0.33247 | -0.00006 | 0.10104 |
| AHRR | cg16896326 | 0.00123 | 0.45087 | 0.00172 | 0.35093 | 0.00016 | 0.0798 |
| AHRR | cg04879308 | 0.00713 | 0.26178 | -0.00656 | 0.36148 | 0.00017 | 0.63265 |
| AHRR | cg17668415 | 0.00054 | 0.84341 | -0.00249 | 0.42015 | 0.00008 | 0.62555 |
| AHRR | cg14714797 | 0.0043 | 0.41486 | -0.00441 | 0.45998 | 0.00055 | 0.06673 |
| AHRR | cg12961784 | 0.00166 | 0.74565 | 0.00416 | 0.47127 | -0.00031 | 0.28926 |
| AHRR | cg14744022 | -0.00089 | 0.65507 | 0.00156 | 0.48644 | -0.00018 | 0.10033 |
| AHRR | cg09874656 | -0.00613 | 0.09824 | 0.00285 | 0.49762 | -0.00026 | 0.21558 |
| AHRR | cg02527419 | 0.00018 | 0.90119 | 0.00108 | 0.50324 | 0.00012 | 0.13223 |
| AHRR | cg08238319 | -0.03 | 0.29375 | -0.02128 | 0.51049 | 0.00117 | 0.46977 |
| AHRR | cg00401753 | -0.00117 | 0.4594 | -0.00114 | 0.52632 | -0.00006 | 0.49521 |
| AHRR | cg08858540 | -0.00234 | 0.33909 | -0.00168 | 0.54147 | -0.00025 | 0.06643 |
| AHRR | cg25430111 | -0.00072 | 0.57166 | 0.00085 | 0.55494 | -0.00003 | 0.69952 |
| AHRR | cg08916839 | 0.01961 | 0.35491 | 0.01205 | 0.61547 | -0.00103 | 0.39261 |
| AHRR | cg15945600 | -0.00351 | 0.28624 | 0.00174 | 0.63994 | -0.00035 | 0.05689 |
| AHRR | cg16995193 | 0.00211 | 0.2419 | -0.00089 | 0.66448 | 0.00006 | 0.52591 |
| AHRR | cg20310920 | -0.00086 | 0.77783 | 0.00139 | 0.6862 | -0.00021 | 0.23127 |
| AHRR | cg16371648 | 0.00212 | 0.644 | -0.00203 | 0.6975 | 0.00016 | 0.54002 |
| AHRR | cg04286878 | 0.00024 | 0.68866 | -0.00027 | 0.69759 | 0.00004 | 0.29532 |
| AHRR | cg18541609 | -0.00285 | 0.05145 | 0.00061 | 0.71175 | 0.0000 | 0.97282 |
| AHRR | cg01571467 | -0.00192 | 0.31302 | 0.00071 | 0.741 | -0.00001 | 0.90918 |
| AHRR | cg16081854 | -0.00625 | 0.76864 | -0.00744 | 0.75711 | -0.00173 | 0.15139 |
| AHRR | cg07599136 | 0.00599 | 0.76133 | 0.00491 | 0.82613 | -0.00058 | 0.60453 |
| AHRR | cg16325394 | -0.00291 | 0.19312 | -0.00054 | 0.8317 | 0.00008 | 0.50087 |
| AHRR | cg11827403 | 0.00214 | 0.20488 | -0.0004 | 0.83535 | -0.00011 | 0.23927 |
| AHRR | cg13275321 | -0.00185 | 0.47436 | 0.00055 | 0.84951 | -0.00012 | 0.4205 |
| AHRR | cg20554397 | -0.00081 | 0.62041 | -0.0003 | 0.87168 | -0.00018 | 0.05673 |
| AHRR | cg24955955 | 0.00924 | 0.64824 | 0.00367 | 0.87278 | -0.00059 | 0.60458 |
| AHRR | cg27464615 | 0.00362 | 0.12241 | -0.00038 | 0.88579 | 0.00003 | 0.83931 |
| AHRR | cg15179499 | 0.00319 | 0.17975 | 0.00022 | 0.93485 | 0.00007 | 0.59961 |
| AHRR | cg19039843 | 0.00014 | 0.9425 | 0.00004 | 0.98643 | -0.00021 | 0.05975 |
| AHRR | cg19772705 | -0.00123 | 0.42824 | -0.00002 | 0.99214 | 0.00006 | 0.50305 |
| AHRR | cg04023872 | 0.00007 | 0.914 | 0.00001 | 0.99382 | 0.00005 | 0.20798 |
| **F2RL3** | **cg03636183** | **-0.02371** | **<0.00001**** | **-0.08369** | **<0.00001**** | **-0.00101** | **0.00000264**** |
| **F2RL3** | **cg21911711** | **-0.02023** | **<0.00001**** | **-0.04782** | **<0.00001**** | **-0.00066** | **0.00018243**** |
| F2LR3 | cg04259305 | -0.00731 | 0.0026670* | -0.01105 | 0.00006261** | -0.00056 | 0.00004301** |
| F2LR3 | cg10765427 | -0.00439 | 0.065824 | -0.0255 | <.00001** | -0.00049 | 0.00026363** |
| F2LR3 | cg15159987 | -0.00162 | 0.53220834 | -0.02222 | <.00001** | -0.0004 | 0.00669627* |
| F2LR3 | cg13751927 | -0.00121 | 0.33068343 | -0.00226 | 0.11012389 | -0.00039 | 0.00000004** |
| F2LR3 | cg08200625 | -0.00272 | 0.23277549 | -0.00664 | 0.01030501 | -0.00052 | 0.00005438** |
| F2LR3 | cg03046327 | -0.00277 | 0.07728309 | -0.000856 | 0.62992128 | -0.00053 | <.00001** |
| F2LR3 | cg23948016 | 0.00035707 | 0.84611076 | 0.00143 | 0.4919734 | 0.000811 | <.00001** |
| F2LR3 | cg05211836 | -0.00174 | 0.35234909 | 0.00007431 | 0.9719749 | 0.000587 | 0.00000003** |
| F2LR3 | cg08067617 | 0.00039193 | 0.70611878 | 0.00073876 | 0.53010942 | 0.000417 | <.00001** |
| F2LR3 | cg14753355 | 0.00114 | 0.20932198 | -0.0003431 | 0.73940045 | 0.000348 | <.00001** |
| F2LR3 | cg25211934 | 0.00036734 | 0.63894367 | 0.00025187 | 0.77626041 | 0.000277 | <.00001** |
| F2LR3 | cg19006008 | 0.00004897 | 0.96987484 | 0.0025 | 0.08941328 | 0.000439 | <.00001** |
| F2LR3 | cg10256336 | 0.00203 | 0.13185715 | 0.00025554 | 0.86665034 | 0.000629 | <.00001** |
| F2LR3 | cg23344409 | -0.00222 | 0.17263611 | -0.00474 | 0.01020938* | -0.00027 | 0.00355193* |
| F2LR3 | cg14021375 | -0.00047296 | 0.83447342 | 0.00017708 | 0.94489679 | 0.000315 | 0.01409159* |
| F2LR3 | cg03517458 | -0.00052976 | 0.71968119 | 0.00009591 | 0.95422784 | -0.00025 | 0.00300366* |
| F2LR3 | cg15025569 | -0.00128 | 0.66477872 | -0.00795 | 0.01770175* | -0.000067 | 0.68756703 |
| F2LR3 | cg08066035 | 0.00070012 | 0.40419033 | -0.0002013 | 0.83217801 | -0.00008 | 0.09231131 |

* signifies P<0.05 and ** signifies P<0.00028409 (Bonferroni correction)

Supplementary Table 2. Multiple linear regression analysis showing the association of smoking and PM_2.5_ with F2RL3 cg03636183 methylation in the study participants.

| Variable | Model 1 | | Model 2 | |
| --- | --- | --- | --- | --- |
|  | β | P-value | β | P-value |
| Smoking status (Reference: Never) |  |  |  |  |
| Former | -0.02371 | <0.0001 | -0.01052 | 0.0020 |
| Current | -0.08369 | <0.0001 | -0.02449 | <0.0001 |
| P-trend | <0.0001 | | <0.0001 | |
| PM_2.5_ | -0.00101 | <0.0001 | -0.00054 | 0.0046 |
| Sex (Reference: Women) |  |  |  |  |
| Men | -0.00038 | 0.8907 | 0.00594 | 0.0014 |
| Age | -0.00037 | 0.0028 | -0.00022 | 0.0403 |
| Exercise (Reference: No) |  |  |  |  |
| Yes | 0.00036 | 0.8919 | 0.00010 | 0.9660 |
| BMI | 0.00065 | 0.0637 | 0.00044 | 0.1449 |
| Alcohol drinking (Reference: No) |  |  |  |  |
| Former | 0.00443 | 0.5115 | 0.00606 | 0.2924 |
| Current | -0.00536 | 0.2928 | -0.00589 | 0.1850 |
| Second-hand smoke (Reference: No) |  |  |  |  |
| Yes | 0.00477 | 0.2202 | 0.00431 | 0.2038 |
| AHRR cg05575921 methylation | - | - | 0.33759 | <0.0001 |

Supplementary Table 3. Multiple linear regression analysis showing the association of smoking and PM_2.5_ with F2RL3 cg03636183 methylation in the study participants stratified by sex.

| Variable | Men | | Women | |
| --- | --- | --- | --- | --- |
|  | β | P-value | β | P-value |

| Smoking status (reference: Never) |  |  |  |  |
| --- | --- | --- | --- | --- |
| Former | -0.02423 | <0.0001 | -0.01483 | 0.0416 |
| Current | -0.08775 | <0.0001 | -0.06331 | <0.0001 |
| P-trend | <0.0001 | | <0.0001 | |
| PM_2.5_ | -0.00121 | 0.0004 | -0.00071 | 0.0054 |
| Age | -0.00071 | 0.0003 | 0.00005 | 0.7319 |
| Exercise (Reference: No) |  |  |  |  |
| Yes | -0.00050 | 0.9044 | 0.00092 | 0.7637 |
| BMI | 0.00133 | 0.0251 | -0.00012 | 0.7590 |
| Alcohol drinking (Reference: No) |  |  |  |  |
| Former | 0.00505 | 0.5341 | 0.00988 | 0.5202 |
| Current | -0.00681 | 0.2643 | 0.01909 | 0.2104 |
| Second-hand smoke exposure (Reference: No) |  |  |  |  |
| Yes | 0.00437 | 0.4710 | 0.00403 | 0.3922 |

Supplementary Table 4. Multiple linear regression analysis showing the association of smoking and living in PM_2.5_ areas with F2RL3 cg03636183 methylation in the study participants.

| Variable | β | P-value |
| --- | --- | --- |
| Smoking status (Reference: Never) |  |  |
| Former | -0.002390 | <0.0001 |
| Current | -0.08358 | <0.0001 |
| P-trend | <0.0001 | |
| Area (Reference: North) |  |  |
| North-Center | -0.01084 | 0.0037 |
| Center | -0.00958 | 0.0058 |
| South | -0.01703 | <0.0001 |
| P-trend | <0.0001 | |
| Exercise (Reference: No) |  |  |
| Yes | 0.00023 | 0.9311 |
| Sex (Reference: Women) |  |  |
| Men | -0.00048 | 0.8620 |
| Age | -0.00036 | 0.0035 |
| BMI | 0.00074 | 0.0358 |
| Alcohol drinking (Reference: No) |  |  |
| Former | 0.00366 | 0.5793 |
| Current | -0.00530 | 0.2977 |
| Second-hand smoke exposure (Reference: No) |  |  |
| Yes | 0.00467 | 0.2297 |

Supplementary Table 5. Multiple linear regression analysis showing the association of smoking and living in PM_2.5_ areas and F2RL3 cg03636183 methylation in the study participants stratified by sex.

| Variable | Men | | Women | |
| --- | --- | --- | --- | --- |
|  | β | P-value | β | P-value |
| Smoking status (Reference: Never) |  |  |  |  |
| Former | -0.02421 | <0.0001 | -0.01555 | 0.0338 |
| Current | -0.08765 | <0.0001 | -0.06286 | <0.0001 |
| P-trend | <0.0001 | | <0.0001 | |
| Area (Reference: North) |  |  |  |  |
| North-Center | -0.01386 | 0.0199 | -0.00690 | 0.1161 |
| Center | -0.01132 | 0.0398 | -0.00599 | 0.1446 |
| South | -0.02056 | 0.0001 | -0.01199 | 0.0022 |
| P-trend | <0.0001 | | <0.0001 | |
| Exercise (No) |  |  |  |  |
| Yes | -0.00073 | 0.8614 | 0.00093 | 0.7627 |
| Age | -0.00070 | 0.0003 | 0.00005 | 0.7274 |
| BMI | 0.00144 | 0.0161 | -0.00007 | 0.8617 |
| Alcohol drinking (Reference: No) |  |  |  |  |
| Former | 0.00359 | 0.6591 | 0.01166 | 0.4505 |
| Current | -0.00663 | 0.2766 | 0.01808 | 0.2369 |
| Second-hand smoke exposure (Reference: No) |  |  |  |  |
| Yes | 0.00421 | 0.4868 | 0.00387 | 0.0319 |
